# Impact of IPM practices on microbial population and disease development in transplanted and direct-seeded rice

**DOI:** 10.3389/fmicb.2024.1388754

**Published:** 2024-07-30

**Authors:** M. K. Khokhar, Rakesh Kumar, Anoop Kumar, Mukesh Sehgal, S. P. Singh, P. N. Meena, Niranjan Singh, L. K. Acharya, Ajanta Birah, Kartar Singh, R. S. Bana, M. S. Gurjar, Subhash Chander, Manoj Choudhary

**Affiliations:** ^1^ICAR-National Research Centre for Integrated Pest Management, New Delhi, India; ^2^ICAR-NBPGR Regional Station, Jodhpur, India; ^3^ICAR-Indian Agriculture Research Institute, New Delhi, India; ^4^Department of Plant Pathology, University of Florida, Gainesville, FL, United States

**Keywords:** inoculum density, colony-forming units, IPM, *Trichoderma*, *Pseudomonas*, *Fusarium*, direct-seeded and transplanted rice

## Abstract

Integrated pest management (IPM) is a comprehensive approach to managing diseases, focusing on combining various strategies to reduce pathogen populations effectively and in an environmentally conscious way. We investigated the effects of IPM on beneficial microbial populations and its relationship with pathogen populations in both direct-seeded rice (DSR) and transplanted rice (TR) systems. This study demonstrates that IPM practices have significantly higher populations of beneficial microbes, such as *Trichoderma harzianum* and *Pseudomonas fluorescens*, and lower level of the pathogen *Fusarium verticillioides* compared to non-IPM (farmer practices). The average mean population of *T. harzianum* was 6.38 × 10^3^ CFU/g in IPM compared to 3.22 × 10^3^ CFU/g in non-IPM during 2019 in TR at Bambawad. *P. fluorescens* mean population in 2019 was significantly higher in IPM (4.67 × 10^3^ CFU/g) than in non-IPM (3.82 × 10^3^ CFU/g) at the Karnal location in DSR. The *F. verticillioides* populations were significantly lower in IPM fields (9.46 × 10^3^ CFU/g) compared to non-IPM fields (11.48 × 10^3^ CFU/g) during 2017 at Haridwar in TR. Over three years, a significant increase in the populations of beneficial microbes in IPM plots was observed in all three locations of both TR and DSR, highlighting the sustainable impact of IPM practices. Disease dynamics analysis revealed that IPM effectively managed key diseases in both DSR and TR systems, with significant correlations between microbial density and disease severity. A significant positive correlation was recorded between *F. verticillioides* population and bakanae incidence at all three locations. Sheath blight incidence was negatively correlated with *P. fluorescens* population in both TR and DSR. In DSR, bacterial blight and brown spot diseases are reduced with the increased population of *T. harzianum*. Bioagents *T. harzianum* and *P. fluorescens* reduced disease incidence, underscoring the role of beneficial microbes in disease suppression and their importance for sustainable production using IPM practices.

## Introduction

Rice (*Oryza sativa* L.) is an economically important food crop in many developing countries, particularly, in the South Asian region. Approximately 50% of total agricultural land in South Asia is covered by rice-based cropping system. Rice is one of the most important staple food crops in India and many other countries in Asia. The yield potential of rice is severely affected by many biotic stresses, especially by diseases such as bakanae, rice blast, bacterial leaf blight (BLB), sheath blight, and brown spot are important diseases in rice-growing areas of the world (Imfeld and Vuilleumier, 2012; Tanwar et al., 2016). Yield loss due to diseases in rice can be 10–15% in tropical Asia (Savary et al., 2012b). Yield loss to individual disease may vary depending on geographic location and environmental conditions. For example, rice blast can cause yield loss of up to 50% (Asibi et al., 2019), bakanae diseases (13.8%) (Tanwar et al., 2016), sheath blight (50%) (Khoshkdaman et al., 2021) that depends on varietal susceptibility, the degree of infection, and the timing of fungicide application (Oerke, 2006).

Integrated pest management (IPM) is a holistic approach to disease management that emphasizes the use of multiple strategies to manage pest populations in an effective and environmentally sensitive manner (Chatterjee et al., 2021; Hajjar et al., 2023). Integrated management of rice diseases can improve yield by 10–20% of current harvested yield (Savary et al., 2012a). Unlike traditional pest control methods that may rely heavily on chemical pesticides, IPM incorporates a variety of techniques such as biological control, habitat manipulation, modification of cultural practices, and the use of resistant varieties (Veres et al., 2020; Zhang et al., 2022). The goal of IPM is to reduce or eliminate the use of chemical pesticides, thereby minimizing the potential negative impacts on human health, non-target organisms and the environment.

IPM operates on the principle of employing the least possible hazard to human health and the environment, focusing on the long-term prevention of pests or their damage through a combination of tactics (Singh, 2019). Monitoring and correct pest identification are the cornerstones of IPM, ensuring that any actions taken are based on the actual presence of pests and their potential threat to the crop or environment. Biological control, one of the key strategies in IPM, involves the use of bioagents and natural enemies of pests to control pest population (Babendreier et al., 2020; Veres et al., 2020; Chatterjee et al., 2021).

In India, farmers are mostly dependent on the advice of pesticide dealers and usually take 4–6 sprays of pesticides including a tank mix cocktail of pesticides (Tanwar et al., 2016). Excessive uses of pesticides have adverse effects on the environment which reduces the biodiversity of natural enemies and leads to environmental pollution, development of pesticide resistance, resurgence of pest population, and human health hazards (Avis et al., 2008; Imfeld and Vuilleumier, 2012). To get rid of this menace, some new avenues must be investigated. One of the effective ways is to develop eco-friendly, sustainable, and socio-economic acceptable IPM packages (Tanwar et al., 2016).

Biological control is the most important component of integrated pest management strategy (Khatoon et al., 2020; Somasundaram et al., 2020; Harman et al., 2021). Biological control aims to use natural enemies to regulate pest populations to a level where they do not cause yield loss (Ferreira and Musumeci, 2021; Ansari et al., 2023). However, there is a need for more optimal use of biological control at national and international levels. Technical knowledge and skills concerning the production and sustainable utilization of biocontrol or biopesticides in Asia are still inadequate to warrant its widespread adoption. However, the Indian Council of Agricultural Research (ICAR)-National Research Centre for Integrated Pest Management in New Delhi has already developed IPM packages against several destructive pests of various crops, including rice, with an emphasis on mass trapping with pheromones, the use of botanical and biopesticides, pest-resistant crop varieties, and modification of agronomic practices to reduce pest incidence (Tanwar et al., 2016; Sardana et al., 2020).

Normally, biological control tactics use single isolated microbes, such as *Trichoderma, Pseudomonas, Bacillus,* and *Streptomyces* (Kumar and Ashraf, 2017). However, the potential of microbial biological control always depends on significant knowledge of microbial ecology in the rhizosphere (Martínez-Viveros et al., 2010; Araujo et al., 2019). Microbials suppress pathogens in the rhizosphere through a variety mechanisms, including antibiosis, competition for nutrients, parasitism, and biosynthesis of microbial compounds that inhibit pathogens in the field and express beneficial traits (Compant et al., 2019; de Faria et al., 2021).

The application of bioagents affects the survival and carry-over of microbial pathogens from one crop to another (Berendsen et al., 2012). To improve plant growth and soil health, it is important to know the microorganism present in the rhizosphere microbiome and as its function and purpose. Information on the interaction of soil microbial pathogens with *Trichoderma*/*Pseudomonas* and the appearance of diseases, nematodes, and insect pests in the plants is scanty. There is a need to understand the relationship between disease development and inoculum densities, their antagonistic activity and disease dynamics in the field or laboratory to achieve efficient crop protection in rice-based cropping systems.

Rice cultivation encompasses two primary methods: direct-seeded rice (DSR) and transplanted rice (TR). In DSR, rice seeds are directly sown into the field without prior germination in a nursery bed (Farooq et al., 2011). This method offers advantages such as water conservation, reduced labor, and time efficiency, making it particularly suitable for areas with water scarcity. However, direct-seeded rice is susceptible to diseases such as sheath blight, blast, bacterial leaf blight, and tungro disease (Hufnagel et al., 2020). On the other hand, transplanted rice involves raising rice seedlings in nurseries before transplanting them into the main field after a few weeks of growth. This method allows for better weed control, reduces competition among plants, and ensures uniform crop establishment (Rao et al., 2007; Khaliq et al., 2012). If unchecked, the loss due to weed infestation can be as high as 80% in DSR (Matloob et al., 2015). Despite these advantages, transplanted rice is also vulnerable to diseases such as sheath blight, blast, bacterial leaf blight, brown spot and sheath rot (Rao et al., 2007; Chaudhary et al., 2023).

Rice IPM studies are crucial to address the pressing need for sustainable agriculture practices that can enhance food security while minimizing environmental impacts. By focusing on the relationship between microbial populations, disease development, and soil physicochemical properties under IPM, researchers can identify strategies that promote crop health and yield without relying heavily on chemical inputs (Ganie et al., 2021; Ngalimat et al., 2021; Hajjar et al., 2023). IPM research contributes to the development of eco-friendly pest management practices, reduces the reliance on synthetic pesticides, and helps in maintaining or improving soil health. This, in turn, supports biodiversity, enhances the resilience of cropping systems to diseases, and can lead to more sustainable agricultural productivity (Siegel-Hertz et al., 2018). Through a better understanding of IPM’s effects, stakeholders can implement IPM practices that are not only economically viable but also environmentally safe, ensuring the long-term sustainability of agricultural ecosystems.

To explore the role of antagonist microbial populations in soil in relation to diseases in IPM, we conducted a study with the following objectives: (i) monitor the density of *Trichoderma harzianum, Pseudomonas fluorescens,* and *Fusarium verticillioides* in soil as influenced by IPM interventions in DSR and TR; (ii) determine relationships between microbes density and disease incidence; (iii) perform socio-economic studies, and (iv) investigate the effect of IPM and non-IPM practices on soil physiochemical properties in TR and DSR.

## Methods

### Experimental sites

**Figure 1:**
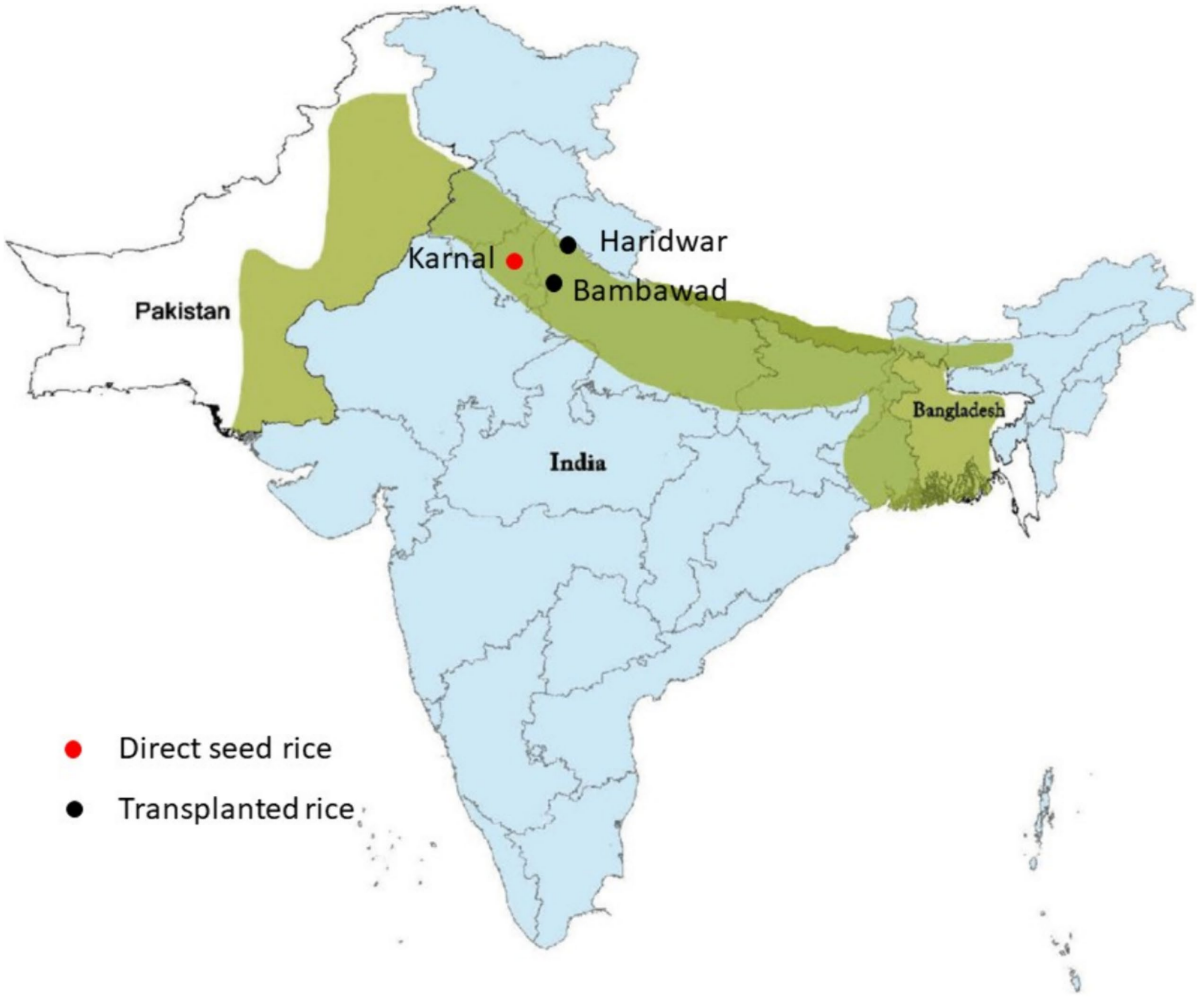
Experiment location site on Indo Gangetic region. The green color represents area in the Indian subcontinent which is mostly irrigated by the Indus and Ganga river and its tributaries. The light blue color is India’s geographical area. Red color dots represent the geographic location of directly seeded rice, while black color dots represent the location for transplanted rice.

In this study, three locations situated in the Indo-Gangetic region of India were selected for study: Bambawad (28.36.46 N, 77.33.18 E) in Uttar Pradesh, Haridwar (29.44.22 N, 78.00.42 E) in Uttarakhand, and Karnal (29.39.40 N, 76.39.01 E) in Haryana. These locations represent key agricultural areas within the region, particularly renowned for their major rice-wheat cropping systems (Figure 1). The selection of these specific locations was aimed at assessing the inoculum density of beneficial microorganisms such as *T. harzianum* and *P. fluorescens,* as well as the presence and dynamics of the pathogen *F. verticillioides*. Each location comprised three fields, each field spanning four acres, providing a comprehensive framework for evaluating microbial populations and disease dynamics across diverse agricultural landscapes.

### IPM module

The IPM module was meticulously implemented across all fields, comprising a series of strategic interventions aimed at promoting sustainable pest and disease management. These interventions included the following:

- Cultivating *Sesbania* in mid-May and incorporating it into the soil 45 days after sowing during land preparation.
- Treating seeds with *T. harzianum* (10^8^ CFU/mL) at 10 g/kg of seeds.
- Immersing seedling roots in a solution of *P. fluorescence* (3.0 × 10^8^ CFU) at 5 mL per liter of water.
- Planting 2–3 seedlings per hill to enhance crop vigor and resilience.
- Applying fertilizers at a balanced ratio of 60 N: 50 P: 40 K kg/ha, supplemented with ZnSO_4_ at 25 kg/ha to address nutrient deficiencies.
- Implementing manual weed management practices to mitigate weed competition.
- Using fungicides/bactericides judiciously based on economic threshold levels (ETL) for disease control. Additionally, *T. harzianum* was applied post-harvest to facilitate the decomposition of rice and wheat residues, promoting soil health and reducing disease carryover.

This comprehensive approach integrates biological, cultural, and chemical methods to minimize pest and disease pressures while promoting sustainable agricultural practices.

### Non-IPM module

The non-IPM module involves agricultural practices implemented regardless of pest and disease occurrences in the field. This approach includes cultivating crops without incorporating Sesbania, indiscriminate use of fungicide and seedling no root dip treatments, planting a single seedling per hill, applying elevated doses of chemical fertilizers (specifically 220 N: 40 P: 0 K kg/ha), neglecting pest monitoring, and resorting to 2–5 chemical pesticide sprays. Although some farmers in non-IPM fields utilized zinc sulfate, it was administered sparingly. This conventional method underscores a reliance on chemical inputs and a lack of proactive pest management strategies, potentially leading to environmental risks and reduced sustainability.

### Soil sample collection

Rhizospheric soil samples were collected from each of three locations. Within each location, 10 representative fields were carefully selected for sampling. This selection process aimed to capture the variability present within each location rice cultivation landscape. Subsequently, within each chosen field, an additional 10 sites (3 × 3 m) were systematically identified and sampled in a “W” shape pattern. This sampling design was intended to account for spatial heterogeneity within the fields, ensuring a robust representation of the soil microbial community across different micro environments.

Upon collection, the soil samples were subjected to a series of preparatory steps to facilitate subsequent analysis. Initially, the collected soil samples were air-dried to preserve their integrity and prevent microbial degradation. Following this, sieving was performed to homogenize the samples and remove any coarse debris or organic matter. These processed soil samples were then carefully stored under appropriate conditions to maintain their quality until further analysis.

### Physio-chemical properties

The collected soil samples underwent thorough analysis to assess a range of physical and chemical properties vital for understanding soil health and fertility. Parameters such as electrical conductivity (EC) were measured to gauge soil salinity levels, while pH levels were determined to ascertain soil acidity or alkalinity. Additionally, the concentration of organic carbon (OC) was evaluated, providing insights into soil organic matter content and overall soil quality (Ozlu and Kumar, 2018).

Furthermore, essential soil nutrients were analyzed, including available nitrogen, phosphorus, and potassium, expressed in kilograms per hectare (kg/ha) (Sarkar and Rakshit, 2023). These nutrients play pivotal roles in supporting plant growth and development, influencing yield potential and crop quality. The availability of micronutrients such as zinc (Zn) was assessed using the diethylenetriaminepentaacetic acid (DTPA) extraction method, highlighting the soil’s capacity to supply critical micronutrients necessary for optimal plant nutrition (Qaswar et al., 2019).

### Isolation of antagonistic microbial and pathogen

Selective isolation of *T. harzianum*, *P. fluorescence*, and *F. verticillioides* was carried out using appropriate selective media using the dilution plate method. In brief, dilution of soil samples was followed by inoculation onto agar plates containing selective media. Dilutions are plated in replicates, allowing for the enumeration of microbial colonies. Colonies are counted after an incubation period, and the colony-forming units (CFUs) are used to calculate microbial population densities per gram of soil. This method enables the isolation and quantification of specific microorganisms (Nash and Snyder, 1962; Larkin, 2015).

Following isolation, the population of each microorganism *T. harzianum*, *P. fluorescens,* and *F. verticillioides* per g of soil was calculated with the help of the following formula.


$$\begin{array}{l} \mathrm{No}.\;\mathrm{of}\;CFU/g\;\mathrm{of}\;\mathrm{rhizosphere}\;\mathrm{soil}\\ =\frac{\mathrm{No}.\;\mathrm{of}\;\mathrm{colonies}/\mathrm{plate}\times \mathrm{Dilution}\;\mathrm{factor}}{\mathrm{Dry}\;\mathrm{weight}\;\mathrm{of}\;\mathrm{the}\;\mathrm{soil}} \end{array}$$

### Identification of *Trichoderma harzianum*, *Pseudomonas fluorescens*, and *Fusarium verticillioides* isolates

Identification of *T. harzianum* relied on established cultural and morphological characteristics outlined by Bissett (1991). Similarly, *F. verticillioides* was identified based on morphological traits using the “Laboratory Manual for Identification of *Fusarium* Species” (Booth, 1977). For *P. fluorescence*, identification was based on colony shape, characteristics, and pigmentation, with further scrutiny under UV light for fluorescence. Both morphological and molecular identification methods were used to ensure accurate identification of all microbial species studied. These comprehensive approaches encompassing cultural, morphological, and molecular techniques enhance the reliability and precision of microbial identification, essential for understanding their roles within soil ecosystems and agricultural systems.

### Disease dynamics and disease rating

Disease ratings for bacterial blight and brown spot were recorded following the well-established 0–9 scale outlined by IRRI (2013). Within each randomly selected plot, measuring 1 square meter in area, approximately 10 hills were selected in a randomized manner. Within each chosen hill, 2–3 leaves were carefully assessed and graded on the 0–9 scale, taking into account the percentage of leaf damage observed. The percent disease index was subsequently calculated based on the methodology described by Horsfall and Cowling (1978). Sheath blight percent incident was recorded for each square meter area. The present disease incidence was determined using the following formula:


$$\begin{array}{l} \mathrm{Percent}\;\mathrm{disease}\;\mathrm{index}\\ =\frac{\mathrm{Sum}\;\mathrm{of}\;\mathrm{all}\;\mathrm{individual}\;\mathrm{disease}\;\mathrm{ratings}}{\mathrm{Total}\;\mathrm{numbers}\;\mathrm{of}\;\mathrm{plants}\;\mathrm{assessed}\times \mathrm{Maximum}\;\mathrm{rating}\;\mathrm{scale}}\times 100 \end{array}$$

Diseases severity and incidence were recorded from the starting of symptoms to the harvesting of the crop.

### Diseases correlation with microbial populations

To elucidate the relationship between disease incidence and soil microbial population, disease severity was correlated with the inoculum density of *T. harzianum*, *P. fluorescens*, and *F. verticillioides*. This correlation analysis aimed to unveil potential associations between microbial abundance and disease manifestation within the experimental fields of both IPM and non-IPM systems. By employing standard procedures, simple correlation and linear regression analyses were conducted to quantitatively assess the strength and direction of these relationships (Gomez and Gomez, 1984). The comprehensive analysis of these relationships sheds light on the intricate interplay between soil microbial populations and disease dynamics, offering valuable insights for sustainable disease management strategies in agricultural systems.

### Socio-economic analysis

In addition to agronomic assessments, a comprehensive socio-economic analysis was conducted to evaluate the economic viability of different agricultural practices. Detailed records of inputs applied in the fields and corresponding grain yields were thoroughly maintained to calculate the benefit–cost ratio (total return divided by total cost). The total cost estimation encompassed material expenses alongside labor costs for various activities, including land preparation, nursery establishment, seedling transplantation, fertilizer application, manual and mechanical weeding, pesticide application, and harvesting. This holistic approach allowed for a thorough examination of the economic implications associated with different agricultural interventions, aiding in informed decision-making for farmers and policymakers alike.

### Data analysis

Disease incidence and inoculum density were computed as the mean values derived from assessments conducted across 10 sites within each field. This approach ensured robust and representative data for subsequent analysis. Data were analyzed for normal distribution. The collected data statistical analysis using the R software version 4.02. A one-way analysis of variance (ANOVA) was used to examine potential differences among treatment groups, with *post-hoc* analysis performed using the least significant difference (LSD) method. Significance levels were set at a *p*-value of ≤0.05, for determining the statistical significance of observed differences.

Plants/plant parts/ microbes used in the present study comply with international, national, and institutional guidelines.

## Results

### Inoculum density of microbial and pathogen in TR at Bambawad

**Figure 2:**
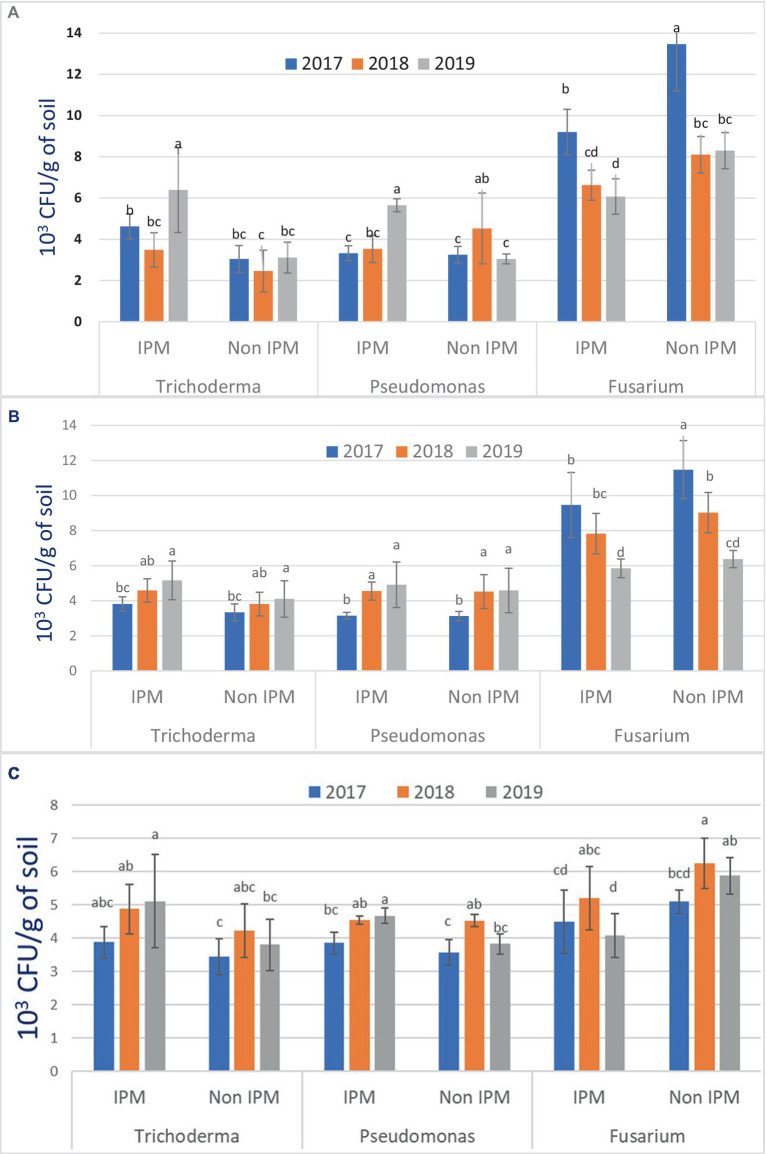
Population density of microbial and pathogens (CFU/g soil) in IPM and non-IPM rice fields at **(A)** Bambawad, **(B)** Haridwar, and **(C)** Karnal. Error bars represent the standard error of the mean. Same letters on graph bars are significantly not different (*p* > 0.05) based on LSD *post-hoc* analysis with use of same bioagents in IPM and non-IPM. Data presented here are an average of 10 sampling locations.

The density of *T. harzianum* was higher in plots under IPM compared to non-IPM fields at Bambawad in TR. A higher number of CFU of *T. harzianum* was recorded (8.40 × 10^3^ CFU/g) in August 2019 followed by September of the same year (7.80 × 10^3^ CFU/g), while the lowest density of *T. harzianum* was recorded in October 2018 (2.65 × 10^3^ CFU/g). Mean population of *T. harzianum* was low (3.49 × 10^3^ CFU/g) in 2018 compared to 2019 (6.38 × 10^3^ CFU/g) in IPM practices (Figure 2A). *T. harzianum* mean population (3.22 × 10^3^ CFU/g) in 2019 was significantly lower in non-IPM than in IPM (*p* < 0.05). During 2017–2019, the population of *P. fluorescens* was initially high in IPM at Bambawad (3.80, 4.30, and 6.10 × 10^3^ CFU/g) compared to non-IPM fields (3.30, 2.00, and 2.80 × 10^3^ CFU/g) but during mid-crop season (August) trend reversed, with CFU recorded higher in non-IPM (3.80, 5.80, and 3.30 × 10^3^ CFU/g) compared to IPM (3.40, 3.65, and 5.40 × 10^3^ CFU/g) in TR at Bambawad (Supplementary Table S1).

Soil-borne pathogen population of *F. verticillioides* had a higher number of CFU in non-IPM plots compared to IPM. In 2017, the mean density of *F. verticillioides* was highest (13.4 × 10^3^ CFU/g) in non-IPM, but later in the third year, CFU decreased significantly to (8.30 × 10^3^ CFU/g soil) (Figure 2A). In IPM, the lowest mean population of *F. verticillioides* was recorded 6.07 × 10^3^ CFU/g. In both IPM and non-IPM, the maximum population of pathogens was recorded during August 2017 (10.3 and 16.4 × 10^3^ CFU/g) and the lowest (6.0 × 10^3^ CFU/g) was recorded October, 2019. A significantly higher population of *P. fluorescens* was observed in 2019 in IPM (5.65 × 10^3^ CFU/g) compared to non-IPM (3.05 × 10^3^ CFU/g) (*p* < 0.05).

### Inoculum density of microbial and pathogen in TR at Haridwar

Throughout the season, IPM plots had a higher population of antagonistic fungus, *T. harzianum*, than non-IPM plots (Figure 2B). The mean populations of *T. harzianum* were numerically higher in IPM in comparison with non-IPM but not significant (*p* > 0.05). In the case of antagonistic bacteria, *P. fluorescence,* population was higher in IPM plots compared to non-IPM plots during all 3 years (Supplementary Table S2). During July 2019, *P. fluorescence* population was 6.75 × 10^3^ CFU/g in IPM in comparison with non-IPM (5.25 × 10^3^ CFU/g). *F. verticillioides* population was significantly lower (9.46 × 10^3^ CFU/g) in IPM during 2017 compared to non-IPM (11.48 × 10^3^ CFU/g) (*p* < 0.05).

### Inoculum density of microbial and pathogen in DSR at Karnal

The density of *T. harzianum* was significantly higher in IPM (5.11 × 10^3^ CFU/g) than non-IPM (3.8 × 10^3^ CFU/g) at Karnal in DSR during 2019 (*p* < 0.05). Higher CFU of *T. harzianum* was recorded in IPM fields during August 2019 (6.70 × 10^3^ CFU/g), while the lowest density was recorded in non-IPM (2.90 × 10^3^ CFU/g) during October 2017 (Supplementary Table S3). Mean population of *T. harzianum* was lower in 2017 (3.87 × 10^3^ CFU/g) in comparison to 2019 (5.11 × 10^3^ CFU/g) in IPM practices.

*P. fluorescens* population increased consistently over the years in IPM practices. It was 3.85 × 10^3^ CFU/g in 2017, which increased to 4.67 × 10^3^ CFU/g in 2019. In non-IPM, highest mean population was recorded in 2018 (4.52 × 10^3^ CFU/g). The mean population of *P. fluorescens* was higher in IPM (4.67 × 10^3^ CFU/g) at the end of the experiment in 2019 compared to non-IPM (3.82 × 10^3^ CFU/g). The density of *F. verticillioides* in IPM was numerically lower in comparison with non-IPM fields over the years. *F. verticillioides* population was significantly lower in the IPM fields (4.07 × 10^3^ CFU/g) compared to non-IPM (5.87 × 10^3^ CFU/g) in 2019. The inoculum density of *F. verticillioides* was lowest in IPM (3.10 × 10^3^ CFU/g) during October 2019 while highest (7.10 × 10^3^ CFU/g) in non-IPM fields during September 2018. In IPM system at Karnal, the highest population of the pathogen was recorded in September, 2018 (6.25 **×** 10^3^ CFU/g) (Figure 2C).

### Percent change in the microbial population in IPM and non-IPM fields in 2019 over 2017

**Figure 3:**
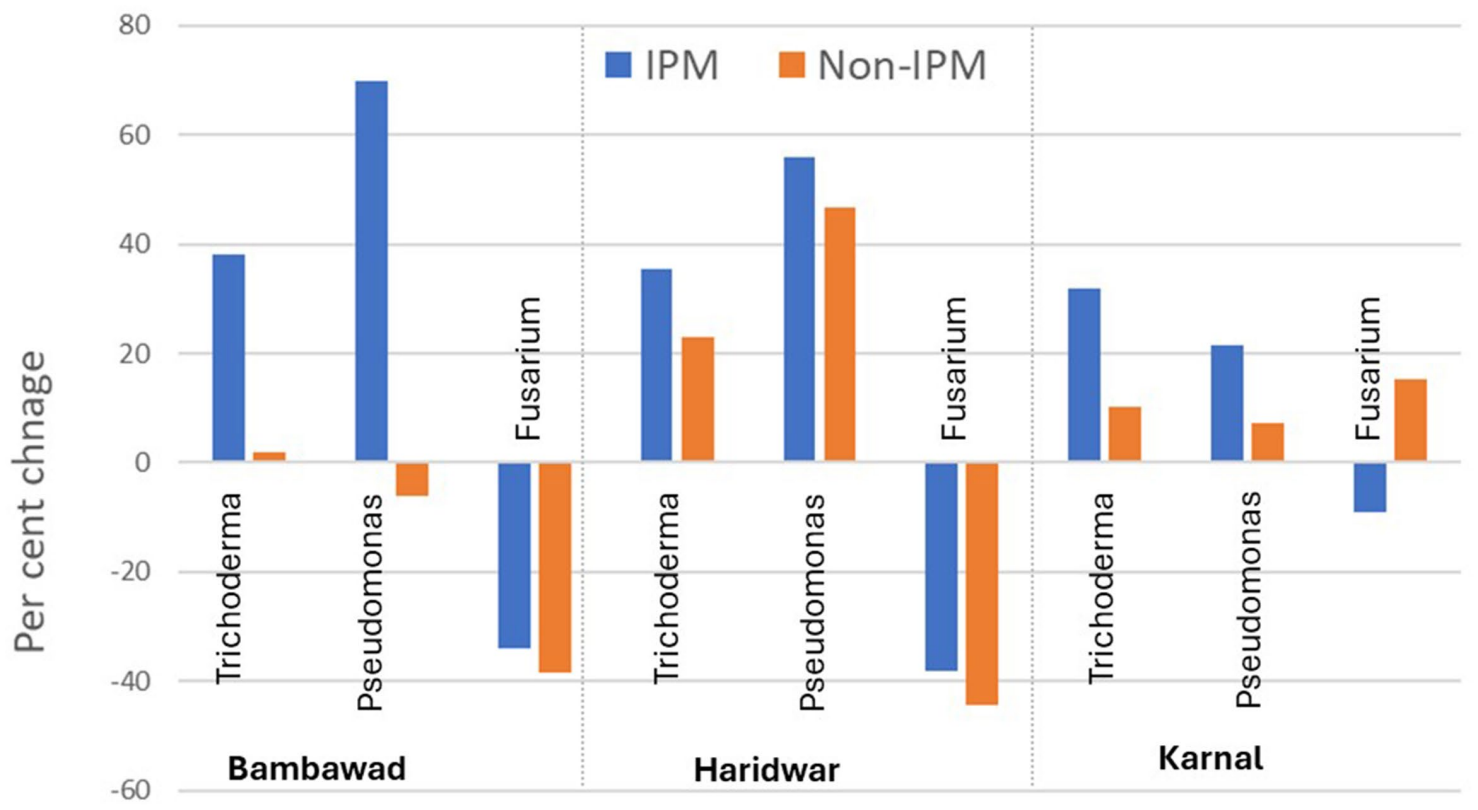
Effect of IPM intervention on percent increase/decrease in microbial population in 2019 over 2017 in IPM and non-IPM fields. Data presented here are an average of 10 samples per location.

At the TR location at Bambawad, an increase of 38.1% in CFU counts of *T. harzianum* was recorded over the years in IPM fields, while in non-IPM only a 2.0% increase was recorded. There was a 69.9% increase over the years for *P. fluorescens* under IPM, while in non-IPM it decreased by 6.2% (Figure 3). Over the years, the percent reduction in *F. verticillioides* population was higher in non-IPM fields (−38.4%) compared to IPM fields (−33.9%). The mean population of *F. verticillioides* in IPM fields was lower by 53.45% over non-IPM fields in TR at Bambawad. Similarly, 49% lower population was TR at Haridwar (Supplementary Tables S1, S2).

At Karnal in DSR, the mean percent increase of *P. fluorescens* over the years under IPM was 21.4% and in non-IPM, it was only 7.0% (Figure 3). An increase of 31.9% in CFU of *T. harzianum* was recorded during 2019 over 2017 in IPM, whereas only a 10.1% increase was recorded in non-IPM. Over the year, reduction in the *F. verticillioides* population was 9.1% in IPM compared to 15.2% increase in non-IPM (Figure 3). The overall reduction of *F. verticillioides* in IPM over non-IPM was 27.33% in DSR (Supplementary Table S3).

### Inoculum density and disease development relationship at Bambawad in TR

Disease incidence of bakanae was 4.0 to 30.4% in IPM and 8.0 to 32.4% in non-IPM fields (Supplementary Table S4). The maximum incidence of BLB was 16.4 and 34.9% in October 2019 in IPM and non-IPM fields, respectively. The maximum disease incidence of bakanae was 30.4% during September 2017 in IPM and 32.4% during October 2019 in non-IPM. Almost a similar trend was recorded for sheath blight and brown spot in non-IPM but in the IPM field’s incidence of both diseases was in traces (Supplementary Table S4).

**Figure 4:**
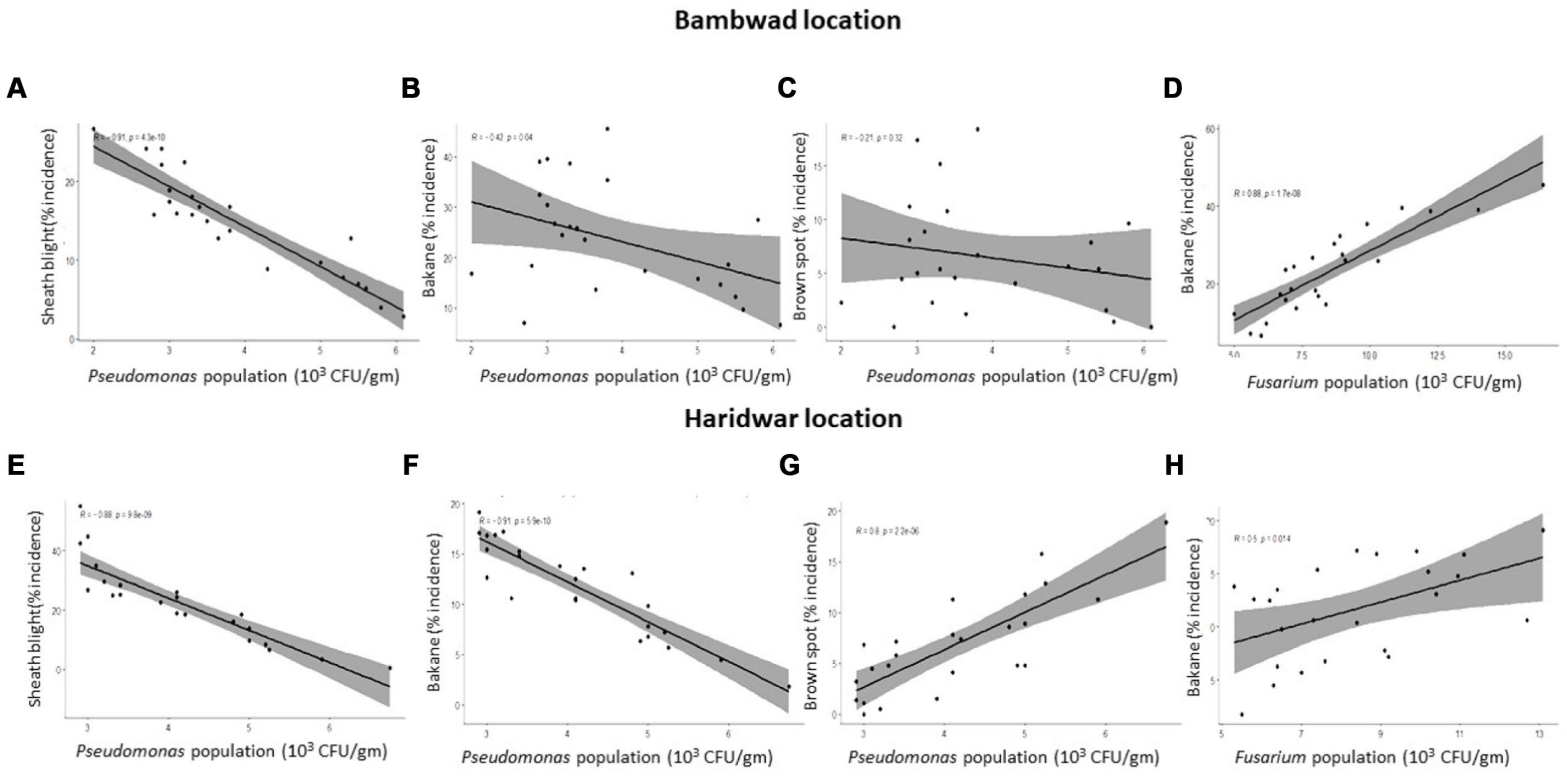
Correlation between disease incidence and microbial population in soil in transplanted rice at Bambawad **(A–D)** and Haridwar **(E–H)**.

The inoculum density of *F. verticillioides* (per g of soil) and bakanae disease incidence in TR at Bambawad showed a significant positive correlation (*r* = 0.88) (Figure 4D), indicating the role of pathogens in disease development. *P. fluorescens* population had a strong negative linear correlation with the disease severity of sheath blight (*r* = 0.91), while a weak negative correlation with bakanae (*r* = 0.42) severity at Bambawad in TR (Figures 4A,B).

### Inoculum density and disease development at Haridwar in TR

Sheath blight was a major disease at Haridwar in TR, with a maximum incidence of 26.6% in October 2017 in IPM and 54.9% in October 2019 in non-IPM. Bacterial leaf blight was also a major disease at Haridwar with a highest incidence of 47.2% during October 2019 in non-IPM fields and 28.70% incidence in IPM fields during September 2018. Disease incidence of brown spot and bakanae was low in both IPM and non-IPM (Supplementary Table S4).

*P. fluorescens* inoculum density was negatively correlated with sheath blight incidence (*r* = 0.88) and bakanae diseases (*r* = 0.91), while brown spot incidence (*r* = 0.80) was positively correlated (Figures 4E–G). A moderate positive correlation (*r* = 0.50) was also observed between bakanae incidence and *F. verticillioides* inoculum density (Figure 4H). However, there was no correlation between *T. harzianum* and bakanae incidence (data not shown).

### Inoculum density and disease development at Karnal in DSR

A varied disease development pattern was observed in DSR at Karnal in comparison with TR at Bambawad and Haridwar. Brown spot and BLB were major diseases recorded at Karnal. Initially, disease incidence was low which built up gradually with the advancement of cropping season. Disease incidence of BLB was high in non-IPM (70.2%) and IPM fields (56.1%) in October 2018, while low incidence was observed in July in both IPM and non-IPM. Highest disease incidence of brown spot was 36.7% in IPM compared to 56.3% incidence in non-IPM (Supplementary Table S4).

**Figure 5:**
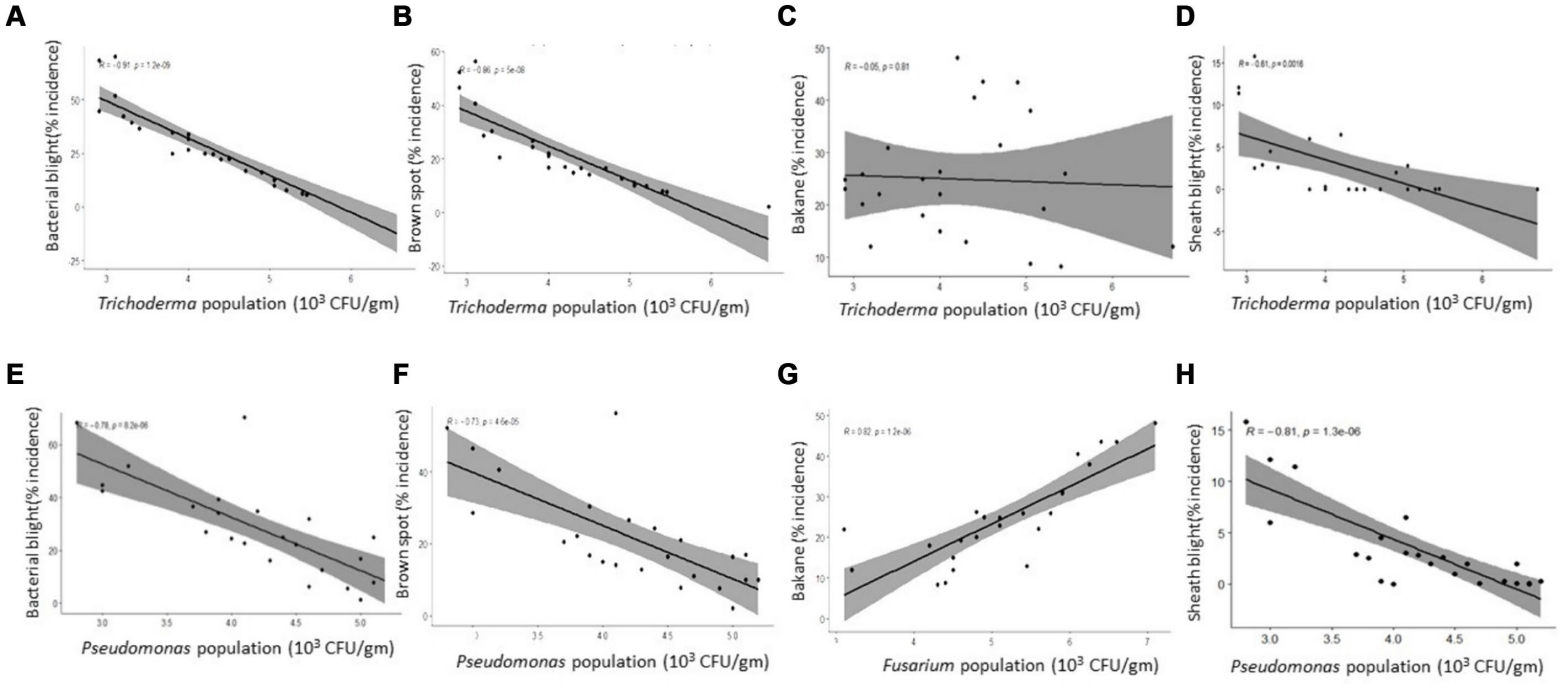
Correlation between disease incidence and microbial population in soil at direct seed rice at Karnal. **(A)** *Trichoderma harzianum* with bacterial blight; **(B)** *T. harzianum* with brown spot; **(C)** *T. harzianum* with bakane; **(D)** *T. harzianum* with sheath blight; **(E)** *Pseudomonas flourescens* with bacterial blight; **(F)**. *P. flourescens* with brown spot; **(G)** *Fusarium verticillioides* with bakane; **(H)** *Pseudomonas flourescens* with sheath blight.

*T. harzianum* inoculum density had a strong significant negative linear relationship with disease severity of BLB (*r* = 0.91), brown spot (*r* = 0.86), and sheath blight (*r* = 0.81) (Figures 5A,B,D) while no correlation with bakanae diseases (Figure 5C). The inoculum density of *P. fluorescens* was also negatively correlated with disease severity of bacterial blight (0.78), brown spot (*r* = 0.73), and sheath blight severity (*r* = 0.81) (Figures 5E,F,H) while no correlation with bakanae diseases (*r* = 0.15, *p* = 0.47).

A strong positive correlation (*r* = 0.81) was observed between bakanae incidence and inoculum density of the *F. verticillioides* (Figure 5G). From these findings, it can be concluded that the CFU density of *T. harzianum, P. fluorescens,* and *F. verticillioides* in soil influenced the severity of rice diseases.

### Effect of physio-chemical properties in IPM and non-IPM field

Soil was neutral to slightly alkaline in all locations; however, pH was high in DSR compared to TR (Table 1). EC of the soil suggested that soil in the three locations of study is non-saline and was suitable for rice cultivation. The observations showed an increase in soil organic carbon (54.05, 10.59, and 33.77%), potash (97.14 41.54, and -23.76%), and zinc (144.53, 100%, and 17.51) under IPM-treated fields in Bambawad, Haridwar, and Karnal in 2019 compared to 2017 (Table 1). However, pH (0.71, 0.50, and 0.03) and EC (0.03, 0.03, and 0.06) were decreased in all three locations at Bambawad, Haridwar, and Karnal, respectively. Phosphorus showed a decline by 56.04 and 39.73% at Bambawad and Haridwar, respectively, while at Karnal it was increased by 48.29% in IPM fields.

**Table 1:** Percent change over the years in physicochemical properties of soil in IPM and non-IPM fields between 2017 and 2019.

Location	Soil properties	2017		2019		Change over the years	
		IPM	non-IPM	IPM	non-IPM	IPM	non-IPM
Bambawad	pH	7.81	7.46	7.10	7.38	−0.71	−0.08
	EC (dS/m)	0.95	0.90	0.92	0.98	−0.03	0.08
	Organic carbon (%)	0.37	0.45	0.57	0.49	54.05%	8.89%
	Available N kg ha-^1^	146.0	125.2	105.4	125.0	−27.81%	−0.16%
	Available K kg ha^−1^	127.32	120.4	251.0	226.0	97.14%	87.71%
	Available P kg ha^−1^	40.95	44.3	18.0	14.4	−56.04%	−67.49%
	Available Zn kg^−1^	1.28	1.70	3.13	1.60	144.53%	−5.88%
Haridwar	pH	7.50	7.65	7.00	7.40	−0.50	−0.25
	EC (dS/m)	0.15	0.17	0.12	0.25	−0.03	0.08
	Organic carbon (%)	0.85	0.63	0.94	0.71	10.59%	12.70%
	Available N kg ha-^1^	215.0	198.1	206.0	212.0	−4.19%	7.02%
	Available K kg ha^−1^	104.0	96.3	147.2	114.0	41.54%	18.38%
	Available P kg ha^−1^	148	88.8	89.2	75.6	−39.73%	−14.86%
	Available Zn kg^−1^	1.40	1.05	2.80	2.10	100.00%	100.00%
Karnal	pH	8.31	8.62	8.28	8.42	−0.03	−0.20
	EC (dS/m)	0.18	0.57	0.12	0.34	−0.06	−0.23
	Organic carbon (%)	0.77	0.33	1.03	0.39	33.77%	18.18%
	Available N kg ha-^1^	167	146	376	267	125.15%	82.885
	Available K kg ha^−1^	442	178	337	101	−23.765	−43.26%
	Available P kg ha^−1^	43.9	15.5	65.1	5.97	48.29%	−61.48%
	Available Zn kg^−1^	4.34	0.40	5.10	0.74	17.51%	85.00%

(−) = percent decrease over the year.

### Socio-economic analysis of IPM and non-IPM fields

Socio-economic studies indicated that higher mean yields were obtained in IPM (40.63 q/ha, 58.98 q/ha, and 55.30 q/ha) compared to non-IPM (36.23 q/ha, 47.98 q/ha, and 53.1 q/ha) in Bambawad, Haridwar, and Karnal, respectively. Three-year pooled benefit–cost ratio was higher (3.19, 3.92, and 4.46) in IPM trials at Bambawad, Haridwar, and Karnal compared to non-IPM (2.29, 2.87, and 3.23), respectively. In the case of IPM, the total cost was Rs 34,013/−, Rs. 31,881/−, and Rs. 29,801/− per ha, respectively, which was low due to reduction in number of pesticide applications compared to non-IPM (Rs. 40,800/−, Rs.34,934/−, and Rs. 40,281/− per ha, respectively) at Bambawad, Haridwar, and Karnal, respectively. IPM fields also resulted in higher net returns of Rs. 74,274/−, Rs. 94,169/−, and Rs. 102,231/− per ha, respectively, compared to non-IPM (Rs. 52,551/−, Rs. 65,374/−, and Rs. 86,049/− per ha) at Bambawad, Haridwar, and Karnal, respectively (Table 2).

**Table 2:** Yield and benefit–cost ratio in cv. Pusa Basmati 1,121 in IPM and non-IPM fields during 2017 to 2019.

Location	Year	Cost (Rs)		Total return (Rs)		Net return (Rs)		Yield (q)		B: C ratio	
		IPM	non-IPM	IPM	non-IPM	IPM	non-IPM	IPM	non-IPM	IPM	non-IPM
Bambawad	2017	32,760	38,451	117579.8	94888.75	84819.8	56437.75	44.12	36.85	3.59	2.47
	2018	34,996	44,216	107612.7	95867.25	72616.7	51651.25	40.38	37.23	3.08	2.17
	2019	34,285	39,735	99,671	89,301	65,386	49,566	37.4	34.68	2.91	2.25
	Mean	34,013	40,800	108,287	93,352	74,274	52,551	40.63	36.23	3.19	2.29
% Change over non-IPM		(−) 16.0		(+) 16.0		(+) 41.3		(+) 12.1			
Haridwar	2017	32,797	33,862	1,67,170	1,23,088	1,34,373	89,226	73	53.7	5.09	3.63
	2018	30,675	34,225	83,444	71,463	52,769	37,238	48.5	44	2.72	2.08
	2019	32,170	36,716	127,535	106,375	95,365	69,659	55.45	46.25	3.96	2.89
	Mean	31,881	34,934	105,489	88,919	94,169	65,374	58.98	47.98	3.92	2.87
% Change over non-IPM		(−) 8.74		(+) 18.6		(+) 44.0		(+) 22.9			
Karnal	2017	30,735	28,385	115,562	107,550	84,827	79,165	53.75	50.0	3.76	3.78
	2018	28,085	44,358	159,600	154,755	131,515	110,397	56	54.3	5:69	3.48
	2019	30,585	48,100	120,937	118,250	90,352	70,150	56.25	55.0	3.95	2.45
	Mean	29,801	40,281	132,033	126,851	102,231	86,571	55.3	53.1	4.46	3.24
% Changeover non-IPM		(−) 26.0		(+) 4.0		(+) 18.09		(+) 4.14			
*t*-test (Mean ± SD)**		28665.5 ± 48	32574.9 ± 718	101296.7 ± 914	78,455 ± 18300.4	72631.1 ± 66	45880.2 ± 1,295	38.0 ± 3.4	30.5 ± 7.1	3.5 ± 0.6	2.0 ± 1.1

Figures presented in parentheses are total return (income); total cost included labor cost for land preparation, nursery sowing, puddling, transplanting, fertilizer application, hand weeding, pesticide application, etc. and material cost such as seed, fertilizer, pesticides, biocontrol agents, and irrigation;*percent increase (+) or decrease (−) over non-IPM; ** significantly different at both 5 and 1% level of significance; B:C – Benefit–cost. Rates of Rice (Rs per q) were: 3150, 3,500, and 2,850 and 3,000, 3,350, and 2,700 in IPM and non-IPM during 2017, 2018, and 2019, respectively. An average rate of 10 years (IPM Rs. 2,665/− and Non-IPM Rs. 2,575/−) was taken.

## Discussion

This is probably the first study that describes the relationships between the microbial inoculum density and disease incidence and the effect of IPM interventions on the soil population of *T. harzianum, P. fluorescens,* and *F. verticillioides* in DSR and TR at farmers’ fields at three locations in major rice-wheat growing region of India. Information on this is crucial for the assessment of disease risk and the development of an integrated disease management module.

Soil sample analysis of three locations, two in TR and one in DSR during 2017–2019 in rice crop, indicated significantly higher population of antagonistic fungi, *T. harzianum,* and antagonistic bacteria, *P. fluorescence,* in IPM compared to non-IPM fields. Maximum population density of *T. harzianum* and *P. fluorescence* was observed in mid-season in August–September months (2017–2019). At the time of sowing and transplanting of the rice seedlings, the count of *T. harzianum* was low in both TR and DSR locations, and the population of *T. harzianum* increased till the flowering stage (August–September). Higher population of *T. harzianum* was recorded in August–September during flowering coinciding with changes from vegetative to reproductive phase due to changes in exudation pattern. The present investigation recorded an increase in the population of *T. harzianum* and *P. fluorescens* with increasing plant age up to August–September which was possibly due to the creation of spermosphere at the initial growth period and subsequently changed the rhizosphere in the growing phase of the crop (Larkin et al., 2011; Larkin and Tavantzis, 2013).

The microbial population of *P. fluorescence* before transplanting was high in IPM at Bambawad and Haridwar compared to non-IPM fields. However, during the mid-crop season, the trend reversed, with CFU recorded higher in non-IPM compared to IPM at Bambawad and Haridwar in TR, while the microbial density of *P. fluorescens* was higher in DSR at the time of sowing at Karnal. The present study indicated that host plant, soil type, agroecology, microclimate, and soil microbial communities had a combined effect on the diversity of fluorescent pseudomonads which induced population build-up. In future studies, use of a dual consortium of bioagents may be used for better disease management in agroecosystems (Sarkar and Rakshit, 2021, 2023).

In the present study, the abundance of inoculum density of *T. harzianum* and *P. fluorescens* was affected by crop management practices in all 3 years. Earlier researchers showed that indigenous *T. harzianum* species were known to have a greater tolerance for pesticides than other soil microorganisms (Bonanomi et al., 2010). In our study, the inoculum density of *T. harzianum* and *P. fluorescens* was found higher in fields under IPM than non-IPM during 2017–2019, which might be due to the different management practices in conventional farms due to the application of fungicides and synthetic fertilizers. In this perspective, the dynamics of beneficial microbial communities can be strongly influenced by soil interactions, agricultural management practices, and moisture (Mavrodi et al., 2018).

In the present study, the population density of *F. verticillioides* was higher in non-IPM fields compared to IPM in all three locations. Present findings are in line with the earlier findings in various cropping systems (Pullman, 1982; Harris et al., 1993; Mendes et al., 2011; Bhatt et al., 2012). For disease management, it is desirable to reduce pathogen inoculum density to a desired low level. The information generated from this study would help to decide on the nature of disease management strategies for rice crops in both TR and DSR systems. In general, an increase in CFU counts of both tested bioagents *T. harzianum* and *P. fluorescens* was recorded over the years. DSR recorded a 9.2% reduction in *F. verticillioides* population in IPM over the years. Interestingly in non-IPM, the *F. verticillioides* population also decreased significantly, which may be due to the high number of pesticide applications in non-IPM fields compared to IPM fields.

During the 3 years of this study, the disease spectrum was not uniform and did not follow a specific trend. Bakanae and BLB in Bambawad, BLB, sheath blight, and bakanae in Haridwar, and brown spot in Karnal were the major diseases recorded during the period. In general, lower disease incidence was reported in IPM in comparison with non-IPM fields. Increased disease severity in non-IPM fields may be associated with increased nitrogen fertilization and dense canopy, while regular addition of organic amendments and biocontrol agents might have led to induced disease suppression in IPM fields (Bulluck and Ristaino, 2002; Davis et al., 2010). *Trichoderma* and *Pseudomonas* not only promote plant growth and development (Wintermans et al., 2016) but also prevent the colonization of plant pathogens and induce plant defense resistance to biotic and abiotic stresses (Rais et al., 2016; de Faria et al., 2021; Kaur et al., 2022).

Our study demonstrated that the incidence of rice diseases in DSR and TR decreased, whereas the population of antagonistic *T. harzianum* and *P. fluorescens* increased in IPM fields. However, the population of *T. harzianum* and *P. fluorescens* was not directly correlated with disease suppressiveness in all diseases. As previously reported, the incidence of southern blight in maize has decreased (Longa et al., 2009; Larkin et al., 2011). A strong and weak correlation between inoculum density and disease incidence in our study under field conditions reflects combined effect of hosts, cultural practices, soil type, environmental conditions, host resistance, and virulence variation of pathogenic isolates prevailing in the soil. A similar type of results was also reported earlier (Joaquim, 1991; Harris et al., 1993; Weller et al., 2002). In the present study, linear regression analysis between inoculum density and disease development gave both positive and negative correlations in TR and DSR.

Soil pH was reduced in both IPM and non-IPM fields studied at three locations (0.71 in Bambawad, 0.50 at Haridwar, and 0.04 in Karnal). EC of the soil suggested that in all three locations, soil was non-saline and was suited for rice cultivation. It was observed that organic carbon increased in IPM and non-IPM fields at all the locations. Improvement of macronutrients (K) and micronutrient Zn was observed over the years in IPM fields at Bambawad and Haridwar. Available N in soil improved significantly in both IPM and non-IPM fields which might have increased the incidence of foliar diseases such as BLB, brown spot, and sheath blight. In IPM, *Sesbania rostrata* was applied as green manure leading to increased microbial activity and organic “C” content and decreased pH and EC of soil which also might have favored increase in propagules of *T. harzianum* and *P. fluorescens* in IPM fields and possibly responsible for the low incidence of diseases in IPM fields. Availability of macronutrients such as N, P, and K were significantly influenced by agriculture practices (Jat et al., 2018; Zhang et al., 2018; Sithole and Magwaza, 2019), which corroborates higher N, P, and K observed during the present study under both IPM and non-IPM fields. Soil properties such as soil organic carbon, macronutrients (N and K), and micronutrients (Zn and Mn) were the important variables that significantly influenced the structure and distribution of microbes. The use of green manuring, organic amendments, biocontrol agents, and need-based application of pesticides in IPM fields influenced the availability of several plant nutrients that improved the physical and chemical conditions of the soil in wetland rice (Tilak, 2004). Our results also corroborate with the findings of Singh (2019), who suggested application of IPM inputs over the years not only improves the soil health and fertility but also helps to manage the rice root-knot nematode, *M. graminicola*.

In our study, socio-economic studies conducted at Bambawad indicated higher yield as well as benefit–cost ratio in IPM compared to non-IPM in TR and DSR. It is mainly due to good agriculture practices and reduction in number of pesticides in IPM. A similar trend of higher yield and B-C ratio in IPM as compared to non-IPM was also observed at Haridwar and Karnal. In all the IPM trials conducted from 2017 to 2019, net return remained higher as compared to non-IPM at all three locations. It is evident from the economic point of view that the application of bioagents along with the adoption of the IPM package resulted in a significant reduction in the disease incidence in IPM fields compared to non-IPM. Green manuring in IPM has favored the availability of nutrients along with N to the crop, which helped in reducing the application of additional N, which could be one of the factors responsible for the low incidence of diseases in IPM fields. This principle could be useful in integrated disease management strategies of many soil-borne pathogens, the incidence of which is dependent on the initial inoculum density.

## Conclusion

This study highlighted the benefits of IPM interventions on soil physicochemical properties and microbial communities. Density of *T. harzianum* and *P. fluorescens* was higher in IPM during 2017–2019, while the inoculum density of *F. verticillioides* was higher in non-IPM fields. Both significant positive and negative correlations were observed between disease incidence and pathogen inoculum density of *T. harzianum, P. fluorescens,* and *F. verticillioides*. Increased levels of soil organic carbon in IPM fields contributed toward good soil health. Socio-economic studies indicated higher yield and benefit–cost ratio in IPM compared to non-IPM. The present study would provide a better understanding of both beneficial microbes and pathogens that could provide a basis for disease risk assessment and the development of biological control strategies for the management of rice diseases. In future, there should be IPM experiments with single bioagent and consortium applications of diseases suppressing agents to explore their effect in disease management.

## Data availability statement

The raw data supporting the conclusions of this article will be made available by the authors, without undue reservation.

## Author contributions

MK: Writing – review & editing, Writing – original draft, Visualization, Validation, Supervision, Software, Resources, Project administration, Methodology, Investigation, Funding acquisition, Formal analysis, Data curation, Conceptualization. RK: Writing – review & editing, Visualization, Validation, Supervision, Software, Resources, Project administration, Methodology, Investigation, Funding acquisition, Formal analysis, Data curation, Conceptualization. AK: Writing – original draft, Visualization, Validation, Supervision, Software, Resources, Project administration, Methodology, Funding acquisition, Formal analysis, Data curation, Conceptualization. MS: Writing – review & editing, Supervision, Investigation. SS: Writing – original draft, Conceptualization. PM: Writing – original draft, Funding acquisition, Formal analysis, Data curation, Conceptualization. NS: Writing – original draft, Project administration, Methodology, Investigation. LA: Writing – review & editing, Writing – original draft, Investigation. AB: Writing – review & editing, Writing – original draft. KS: Writing – review & editing. RB: Writing – review & editing. MG: Writing – review & editing. SC: Writing – review & editing. MC: Writing – original draft, Visualization, Validation, Supervision, Software, Resources, Project administration, Methodology, Investigation, Funding acquisition, Formal analysis, Data curation, Conceptualization, Writing – review & editing.
