# Supplementary material for: Impact of IPM practices on microbial population and disease development in transplanted and direct-seeded rice
Source: Front Microbiol. 2024 Jul 30;15:1388754. doi: 10.3389/fmicb.2024.1388754 (PMC11323746; doi:10.3389/fmicb.2024.1388754)
Supplement: Supplementary file 1 [file Table_1.docx]

Supplementary Table 1. Population density of microbials and pathogens (CFU/g of soil) at Bambawad in IPM and Non IPM rice fields.

| Year | Month and date | Population density of bio-agents | | | | Population density of pathogen | |
| --- | --- | --- | --- | --- | --- | --- | --- |
|  |  | *T. harzianum* (×10^3^) | | *P. flourescens* (×10^3^) | | *Fusarium spp.* (×10^3^) | |
|  |  | IPM | Non IPM | IPM | Non IPM | IPM | NON IPM |
| 2017 | July | 4.90 | 3.25 | 3.80 | 3.30 | 9.90 | 12.25 |
|  | Aug. | 5.30 | 3.55 | 3.40 | 3.80 | 10.30 | 16.40 |
|  | Sept. | 4.40 | 3.30 | 3.00 | 2.90 | 8.70 | 14.00 |
|  | Oct. | 3.90 | 2.10 | 3.10 | 3.00 | 7.90 | 11.20 |
| Mean |  | 4.62 | 3.05 | 3.32 | 3.25 | 9.20 | 13.4 |
| 2018 | July | 4.10 | 3.65 | 4.30 | 2.00 | 6.70 | 8.10 |
|  | Aug. | 4.30 | 2.95 | 3.65 | 5.80 | 7.30 | 9.00 |
|  | Sept. | 2.90 | 1.70 | 3.50 | 5.30 | 6.90 | 8.40 |
|  | Oct. | 2.65 | 1.55 | 2.70 | 5.00 | 5.60 | 6.90 |
| Mean |  | 3.49 | 2.46 | 3.53 | 4.52 | 6.62 | 8.10 |
| 2019 | July | 5.30 | 3.65 | 6.10 | 2.80 | 6.00 | 8.00 |
|  | Aug. | 8.40 | 3.85 | 5.40 | 3.30 | 7.10 | 9.10 |
|  | Sept. | 7.80 | 2.60 | 5.60 | 2.90 | 6.20 | 8.90 |
|  | Oct. | 4.05 | 2.65 | 5.50 | 3.20 | 5.00 | 7.20 |
| Mean |  | 6.38 | 3.22 | 5.65 | 3.05 | 6.07 | 8.30 |
| % change over the year* | | +38.10 | + 2.0 | +69.9 | -6.15 | - 33.9 | -38.4 |

Supplementary Table 2. Population density of microbials and pathogens (CFU/g soil) at Haridwar in IPM and Non IPM fields.

| Year | Month and date | Population density of bio-agents | | | | Population density of pathogen | |
| --- | --- | --- | --- | --- | --- | --- | --- |
|  |  | *T. harzianum* (×10^3^) | | *P. fluorescens* (×10^3^) | | *Fusarium spp.* **(×10^3^)** | |
|  |  | IPM | Non IPM | IPM | Non IPM | IPM | Non IPM |
| 2017 | July | 3.95 | 3.00 | 3.40 | 2.90 | 10.95 | 13.10 |
|  | Aug. | 4.10 | 3.70 | 3.00 | 3.30 | 11.10 | 12.70 |
|  | Sept. | 4.00 | 3.80 | 3.20 | 3.40 | 8.40 | 10.20 |
|  | Oct. | 3.20 | 2.85 | 3.00 | 2.90 | 7.40 | 9.90 |
| Mean |  | 3.81 | 3.33 | 3.15 | 3.12 | 9.46 | 11.48 |
| 2018 | July | 4.75 | 3.90 | 5.00 | 5.00 | 6.50 | 7.60 |
|  | Aug. | 5.30 | 4.40 | 4.10 | 5.20 | 8.40 | 9.20 |
|  | Sept. | 4.60 | 4.10 | 5.00 | 4.80 | 9.10 | 10.4 |
|  | Oct. | 3.70 | 2.85 | 4.10 | 3.10 | 7.30 | 8.90 |
| Mean |  | 4.58 | 3.81 | 4.55 | 4.52 | 7.82 | 9.02 |
| 2019 | July | 4.60 | 3.85 | 6.75 | 5.25 | 5.50 | 7.00 |
|  | Aug. | 5.90 | 4.05 | 4.90 | 5.90 | 6.40 | 6.30 |
|  | Sept. | 6.25 | 5.50 | 4.10 | 4.20 | 6.20 | 6.40 |
|  | Oct. | 3.90 | 3.00 | 3.90 | 3.00 | 5.30 | 5.80 |
| Mean |  | 5.16 | 4.10 | 4.91 | 4.58 | 5.85 | 6.37 |
| % change over the year* | | (+) 35.4 | (+) 22.8 | (+) 55.9 | (+) 46.7 | (-) 38.1 | (-) 44.44 |

Supplementary Table 3. Population density of microbials and pathogens (CFU/g soil) at Direct seeded rice (DSR) location at Karnal in IPM and Non IPM fields.

| Year | Month and date | Population density of bio-agents | | | | Population density of pathogen | |
| --- | --- | --- | --- | --- | --- | --- | --- |
|  |  | *T. harzianum* (×10^3^) | | *P. flourescens* (×10^3^) | | *Fusarium spp.* **(×10^3^)** | |
|  |  | IPM | Non IPM | IPM | Non IPM | IPM | Non IPM |
| 2017 | July | 4.00 | 3.80 | 4.60 | 4.40 | 4.80 | 4.90 |
|  | Aug. | 4.30 | 4.00 | 4.00 | 3.90 | 5.45 | 5.60 |
|  | Sept. | 4.00 | 3.10 | 3.80 | 3.20 | 4.50 | 4.80 |
|  | Oct. | 3.20 | 2.90 | 3.00 | 2.80 | 3.20 | 5.10 |
| Mean | | 3.87 | 3.45 | 3.85 | 3.57 | 4.48 | 5.10 |
| 2018 | July | 5.20 | 4.70 | 5. 10 | 5.00 | 4.60 | 5.90 |
|  | Aug. | 5.45 | 4.90 | 4.90 | 4.30 | 5.75 | 6.60 |
|  | Sept. | 5.05 | 4.20 | 4. 70 | 4.70 | 6.25 | 7.10 |
|  | Oct. | 3.80 | 3.10 | 4.20 | 4.10 | 4.20 | 5.40 |
| Mean | | 4.87 | 4.22 | 4.55 | 4.52 | 5.20 | 6.25 |
| 2019 | July | 5.05 | 4.40 | 5.20 | 4.50 | 4.40 | 6.10 |
|  | Aug. | 6.70 | 4.50 | 5.00 | 4.10 | 4.50 | 6.40 |
|  | Sept. | 5.40 | 3.40 | 4.60 | 3.70 | 4.30 | 5.90 |
|  | Oct. | 3.30 | 2.90 | 3.90 | 3.00 | 3.10 | 5.10 |
| Mean | | 5.11 | 3.80 | 4.67 | 3.82 | 4.07 | 5.87 |
| % change over the year* | | (+) 31.9 | (+) 10.14 | (+) 21.42 | (+) 6.99 | (-) 9.19 | (+)15.67 |

**Supplementary Table 4. Rice diseases incidence at different locations**

| Month of observation | Location -Bambawad | | | | | |
| --- | --- | --- | --- | --- | --- | --- |
|  | Disease- BLB | | | | | |
|  | IPM | | | Non IPM | | |
|  | 2017 | 2018 | 2019 | 2017 | 2018 | 2019 |
| July | 0.00 | 0.00 | 0.02 | 0.00 | 0.00 | 0.20 |
| August | 0.00 | 0.04 | 0.60 | 2.00 | 0.30 | 0.50 |
| September | 5.10 | 2.80 | 1.20 | 12.80 | 16.35 | 12.40 |
| October | 12.00 | 14.00 | 16.40 | 24.20 | 35.00 | 34.90 |

| Month of observation | Location -Bambawad | | | | | |
| --- | --- | --- | --- | --- | --- | --- |
|  | Disease- Sheath Blight | | | | | |
|  | IPM | | | Non IPM | | |
|  | 2017 | 2018 | 2019 | 2017 | 2018 | 2019 |
| July | 0.10 | 0.04 | 0.00 | 2.00 | 2.30 | 0.00 |
| August | 2.03 | 2.30 | 2.80 | 8.20 | 12.00 | 6.40 |
| September | 5.21 | 9.08 | 6.60 | 14.16 | 26.10 | 22.20 |
| October | 11.00 | 19.40 | 17.30 | 26.00 | 30.00 | 26.70 |

| Month of observation | Location -Bambawad | | | | | |
| --- | --- | --- | --- | --- | --- | --- |
|  | Disease- Bakane | | | | | |
|  | IPM | | | Non IPM | | |
|  | 2017 | 2018 | 2019 | 2017 | 2018 | 2019 |
| July | 6.10 | 4.00 | 6.70 | 8.00 | 10.50 | 12.00 |
| August | 9.30 | 7.60 | 9.60 | 12.10 | 14.04 | 16.10 |
| September | 30.40 | 23.60 | 27.60 | 21.03 | 28.80 | 32.40 |
| October | 12.80 | 16.55 | 12.30 | 18.00 | 21.80 | 20.60 |

| Month of observation | Location -Bambawad | | | | | |
| --- | --- | --- | --- | --- | --- | --- |
|  | Disease- Brown Spot | | | | | |
|  | IPM | | | Non IPM | | |
|  | 2017 | 2018 | 2019 | 2017 | 2018 | 2019 |
| July | 0.00 | 0.00 | 0.00 | 0.00 | 0.00 | 0.00 |
| August | 0.10 | 0.04 | 0.00 | 2.40 | 1.66 | 2.40 |
| September | 5.00 | 4.60 | 0.50 | 6.90 | 7.90 | 8.10 |
| October | 15.03 | 17.20 | 1.60 | 17.40 | 15.60 | 16.80 |

| **Month of observation** | **Location -Haridwar** | | | | | |
| --- | --- | --- | --- | --- | --- | --- |
|  | **Disease- BLB** | | | | | |
|  | **IPM** | | | **Non IPM** | | |
|  | 2017 | 2018 | 2019 | 2017 | 2018 | 2019 |
| July | 0.00 | 0.04 | 0.00 | 1.00 | 0.30 | 0.00 |
| August | 3.30 | 0.80 | 0.10 | 2.80 | 6.55 | 4.20 |
| September | 16.20 | 28.70 | 18.90 | 42.20 | 44.10 | 36.80 |
| October | 21.10 | 20.66 | 12.00 | 40.90 | 40.60 | 47.22 |

| **Month of observation** | **Location -Haridwar** | | | | | |
| --- | --- | --- | --- | --- | --- | --- |
|  | **Disease- Sheath Blight** | | | | | |
|  | **IPM** | | | **Non IPM** | | |
|  | 2017 | 2018 | 2019 | 2017 | 2018 | 2019 |
| July | 0.00 | 0.00 | 0.00 | 0.00 | 0.00 | 0.00 |
| August | 4.40 | 2.60 | 1.20 | 14.60 | 25.00 | 3.40 |
| September | 10.80 | 5.80 | 3.50 | 28.36 | 42.40 | 18.60 |
| October | 26.60 | 18.90 | 11.60 | 42.50 | 54.90 | 26.90 |

| **Month of observation** | **Location -Haridwar** | | | | | |
| --- | --- | --- | --- | --- | --- | --- |
|  | **Disease- Brown Spot** | | | | | |
|  | **IPM** | | | **Non IPM** | | |
|  | **2017** | **2018** | **2019** | **2017** | **2018** | **2019** |
| July | 0.00 | 0.00 | 0.00 | 0.00 | 0.00 | 0.00 |
| August | 0.00 | 0.04 | 0.00 | 1.90 | 2.60 | 1.20 |
| September | 0.50 | 3.90 | 0.00 | 7.10 | 8.60 | 7.40 |
| October | 1.03 | 11.30 | 1.50 | 15.20 | 18.40 | 12.90 |

| **Month of observation** | **Location -Haridwar** | | | | | |
| --- | --- | --- | --- | --- | --- | --- |
|  | **Disease- Bakane** | | | | | |
|  | **IPM** | | | **Non IPM** | | |
|  | 2017 | 2018 | 2019 | 2017 | 2018 | 2019 |
| July | 3.00 | 2.60 | 1.80 | 4.50 | 6.80 | 5.70 |
| August | 9.30 | 10.40 | 6.30 | 10.60 | 17.10 | 9.80 |
| September | 8.03 | 3.00 | 8.60 | 15.20 | 13.10 | 13.50 |
| October | 7.20 | 2.60 | 2.60 | 19.10 | 16.90 | 12.60 |

| **Month of observation** | **Location -Karnal** | | | | | |
| --- | --- | --- | --- | --- | --- | --- |
|  | **Disease- BLB** | | | | | |
|  | **IPM** | | | **Non IPM** | | |
|  | **2017** | **2018** | **2019** | **2017** | **2018** | **2019** |
| July | 0.00 | 1.10 | 0.00 | 4.40 | 3.00 | 2.20 |
| August | 4.50 | 5.60 | 1.20 | 12.00 | 16.05 | 12.60 |
| September | 26.80 | 32.50 | 26.30 | 51.90 | 64.30 | 36.60 |
| October | 42.30 | 56.10 | 39.20 | 68.30 | 70.20 | 44.70 |

| **Month of observation** | **Location -Karnal** | | | | | |
| --- | --- | --- | --- | --- | --- | --- |
|  | **Disease- Sheath Blight** | | | | | |
|  | **IPM** | | | **Non IPM** | | |
|  | **2017** | **2018** | **2019** | **2017** | **2018** | **2019** |
| July | 0.00 | 0.00 | 0.00 | 0.00 | 0.00 | 0.00 |
| August | 0.00 | 0.04 | 0.00 | 0.02 | 2.00 | 0.00 |
| September | 0.30 | 2.80 | 0.00 | 2.54 | 6.50 | 2.60 |
| October | 2.90 | 6.00 | 4.50 | 12.10 | 15.80 | 11.40 |

| **Month of observation** | **Location -Karnal** | | | | | |
| --- | --- | --- | --- | --- | --- | --- |
|  | **Disease- Brown Spot** | | | | | |
|  | **IPM** | | | **Non IPM** | | |
|  | **2017** | **2018** | **2019** | **2017** | **2018** | **2019** |
| July | 1.10 | 1.90 | 0.00 | 4.30 | 6.40 | 2.60 |
| August | 4.90 | 7.60 | 2.10 | 16.80 | 12.80 | 14.00 |
| September | 22.10 | 29.00 | 14.00 | 40.60 | 36.90 | 30.60 |
| October | 28.60 | 36.70 | 20.30 | 52.30 | 56.35 | 46.50 |

| **Month of observation** | **Location -Karnal** | | | | | |
| --- | --- | --- | --- | --- | --- | --- |
|  | **Disease- Bakane** | | | | | |
|  | **IPM** | | | **Non IPM** | | |
|  | **2017** | **2018** | **2019** | **2017** | **2018** | **2019** |
| July | 5.20 | 3.70 | 1.20 | 6.00 | 8.30 | 0.00 |
| August | 8.80 | 10.10 | 6.30 | 13.30 | 19.30 | 16.80 |
| September | 11.40 | 14.00 | 10.20 | 29.50 | 36.40 | 23.20 |
| October | 22.10 | 30.90 | 22.90 | 35.90 | 53.80 | 40.10 |
